# Two new long-rostrum cranefly species from the Cretaceous Iberian amber (Diptera, Limoniidae, *Helius*)

**DOI:** 10.1038/s41598-021-91803-1

**Published:** 2021-06-18

**Authors:** Iwona Kania-Kłosok, Wiesław Krzemiński, Antonio Arillo

**Affiliations:** 1 Department of Biotechnology, Institute of Biology and Biotechnology, University of Rzeszów, Zelwerowicza 4, 35–601 Rzeszów, Poland; 2 Institute of Systematics and Evolution of Animals, Polish Academy of Sciences, Sławkowska 17, 31-016 Kraków, Poland; 3 Departamento de Biodiversidad, Facultad de Biología, Ecología y Evolución, Universidad Complutense, 28040 Madrid, Spain

**Keywords:** Evolution, Palaeontology, Taxonomy

## Abstract

First record of the genus *Helius*—long-rostrum cranefly from Maestrazgo Basin (eastern Spain, Iberian Penisula) is documented. Two new fossil species of the genus *Helius* are described from Cretaceous Spanish amber and compared with other species of the genus known from fossil record with particular references to these known from Cretaceous period. *Helius turolensis* sp. nov. is described from San Just amber (Lower Cretaceous, upper Albian) Maestrazgo Basin, eastern Spain, and *Helius hispanicus* sp. nov. is described from Álava amber (Lower Cretaceous, upper Albian), Basque-Cantabrian Basin, northern Spain. The specific body morphology of representatives of the genus *Helius* preserved in Spanish amber was discussed in relation to the environmental conditions of the Maestrazgo Basin and Basque-Cantabrian Basin in Cretaceous.

## Introduction

The family Limoniidae was previously recorded from Spanish amber. Three species were described: *Alavia neli* Krzemiński et Arillo, 2007^1^, *Helius alavensis* Kania, Krzemiński et Arillo, 2016^2^, and *Helius spiralensis* Kania, Krzemiński et Arillo, 2017^3^, all from Peñacerrada I outcrop^1–3^.

In Cretaceous Spanish amber, we find some of the oldest representatives of the genus *Helius*, evidence of the beginnings of the evolution of this group of insects. From the Cretaceous period only six species are known, four from Early Cretaceous^2–5^, and two from Late Cretaceous^6,7^. The oldest representatives of the genus were described based on lower Barremian^8^ inclusions in Lebanese amber, from Hammana-Mdeyrij^4,5^ and Tannourine (Lebanon^4^ and two a little younger from upper Albian of Peñacerrada I (Spain)^2,3^. Two other species were also described from Late Cretaceous, from lower Cenomanian (Tannai village, Myanmar)^7^ and from Turonian (Orapa Diamond Mine (Botswana)^6^. While from younger periods 23 species are known, 13 from Eocene^9–13^, three from Oligocene^14–16^ and seven from Miocene^17–20^ (Table 1). In extant fauna the genus *Helius* is speciose, over 230 extant species of this genus are known and worldwide distributed^21^. It is interesting and still enigmatic that at the beginning of Cretaceous we find species of the genus *Helius* with very elongate rostrum, e.g., Late Cretaceous *Helius botswanensis* Rayner et Waters, 1990^6^ or much older, Early Cretaceous *Helius ewa* Krzemiński, Kania, Azar, 2014^5^ (the oldest known representative of the genus), suggesting on rapid parallel evolution of this insects and Angiospermae at the beginning of Cretaceous period^5^, but additional material is needed to support this hypothesis or finally is needed to explain this problem. Herein, two new peculiar species of *Helius* are described and figured from Spanish amber and characterized by relatively short rostrum.

**Table 1:** List of all fossil species belonging to genus *Helius*, with age and localities.

Species	Age	Type of material	Locality
*Helius miocenicus* Krzemiński, 2002^17^	Miocene	Imprint	Stavropol, Caucasus, Russia
*Helius stavropolensis* Krzemiński, 2002^17^	Miocene	Imprint	Stavropol, Caucasus, Russia
*Helius verticilis* Krzemiński, 2002^17^	Miocene	Imprint	Stavropol, Caucasus, Russia
*Helius* (*Helius*) *ginghai* Wu & Krzemiński, 2019^19^	Miocene	Imprint	Caergen Village, China
*Helius collemus* Podenas & Poinar, 2012^18^	Miocene	Mexican amber	Mexico
*Helius* (*Helius*) *neali* Kopeć, Kania & Krzemiński, 2016^20^	Miocene	Dominican amber	Dominikan Republic
*Helius* (*Helius*) *oosterbroeki* Kopeć, Kania & Krzemiński, 2016^21^	Miocene	Dominican amber	Dominikan Republic
*Helius tenerus* Statz, 1944^15^	Oligocene	Imprint	Rott, Germany
*Helius weigandi* Statz, 1934^14^	Oligocene	Imprint	Rott, Germany
*Helius constenius* Krzemiński, 1991^16^	Oligocene	Imprint	North Montana, USA
*Helius anetae* Kania & Kopeć, 2016^22^	Eocene	Baltic amber	Baltic area
*Helius formosus* Krzemiński, 1993^10^	Eocene	Baltic amber	Baltic area
*Helius linus* Podenas, 2002^11^	Eocene	Baltic amber	Baltic area
*Helius minutus* (Loew, 1850)^9^	Eocene	Baltic amber	Baltic area
*Helius mutus* Podenas, 2002^11^	Eocene	Baltic amber	Baltic area
*Helius pulcher* (Loew, 1850)^9^	Eocene	Baltic amber	Baltic area
*Helius fossilis* Kania, 2014^12^	Eocene	Baltic amber	Baltic area
*Helius gedanicus* Kania, 2014^12^	Eocene	Baltic amber	Baltic area
*Helius similis* Kania, 2014^12^	Eocene	Baltic amber	Baltic area
*Helius hoffeinsorum* Kania, 2014^12^	Eocene	Baltic amber	Baltic area
*Helius* (*Helius*) *edmundi* Krzemiński, 2019^13^	Eocene	Imprint	Isle of Wight, UK
*Helius* (*Helius*) *popovi* Krzemiński, 2019^13^	Eocene	Imprint	Isle of Wight, UK
*Helius* (*Helius*) *vectensis* (Cockerell, 1915)^23^	Eocene	Imprint	Isle of Wight, UK
*Helius botswanensis* Rayner & Waters, 1990^6^	Cretaceous	Imprint	Orapa, Botswana
*Helius krzeminskii* Ribeiro, 2002^7^	Cretaceous	Imprint	Tanai village, Burma
*Helius* (*Helius*) *alavensis* Kania, Krzemiński & Arillo, 2016^2^	Cretaceous	Spanish amber	Álava, Spain
*Helius* (*Helius*) *spiralensis* Kania, Krzemiński & Arillo, 2017^3^	Cretaceous	Spanish amber	Álava, Spain
*Helius lebanensis* Kania, Krzemiński & Azar, 2013^4^	Cretaceous	Lebanese amber	Tannourine, Lebanon
*Helius* (*Helius*) *ewa* Krzemiński, Kania & Azar, 2014^5^	Cretaceous	Lebanese amber	Hammana, Lebanon

## Material and methods

The study was based on two inclusions in Cretaceous amber of Spain. One of these specimens comes from the Upper Albian amber-bearing deposit of Peñacerrada I (Basque-Cantabrian Basin, near the village of Moraza, Province of Burgos). The second one comes from the upper Albian amber-bearing deposit of San Just (Maestrazgo Basin, Utrillas municipality, Province of Teruel).

The first specimen is deposited at the Museo de Ciencias Naturales de Álava, (Vitoria, Spain) and the second one is housed at the Fundación Conjunto Paleontológico de Teruel-Dinópolis (Teruel, Spain).

The specimens were embedded in epoxy resin (EPO-TEK 301) as described^24,25^, which allowed physical protection and optimal study in ventral, lateral and dorsal views.

**Figure 1:**
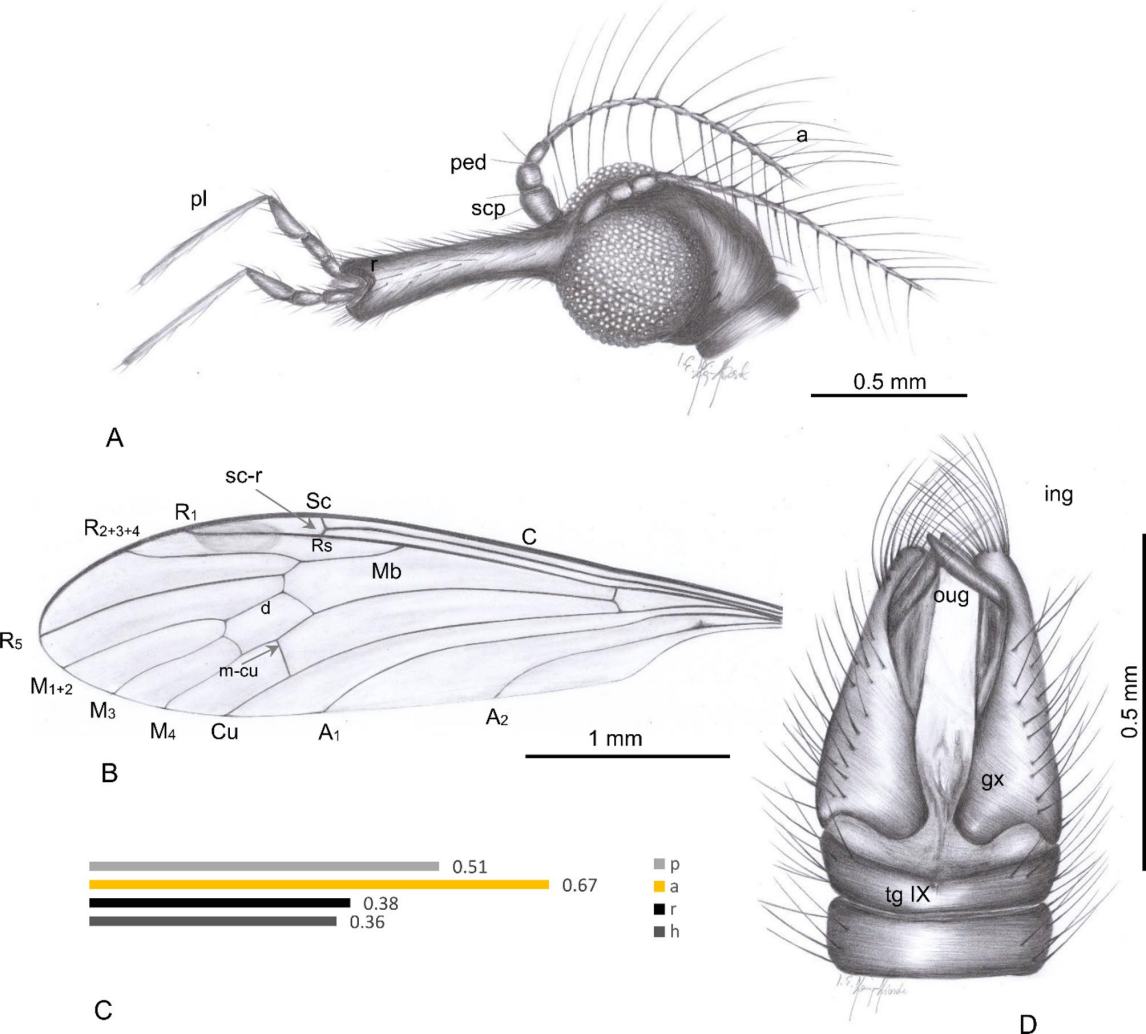
*Helius turolensis* sp. nov. No. 53–10-54 (male), holotype. (**A**) head (lateral view), reconstruction; (**B**) wing; (**C**) the diagram illustrating the relationship between the length of rostrum (r), antenna (a); palpus (p) and head (h); D. hypopygium (dorsal view), reconstruction; Abbreviation of head (h): a—antenna; fl—flagellomeres; pl—palpus; ped—pedicel; r—rostrum; scp—scape.

Both specimens were examined with a Nikon (SMZ25) stereomicroscope Nikon SMZ 1500 equipped with a Nikon DS–Fi1 camera. The measurements were taken with NIS–Elements D 3.0 software. The length of head was measured as length of head capsule excluding rostrum. The length of the discal cell—measurements were given from its posterior edge to the point of connection of vein m-m with vein M_3_. The length of hypopygium was measured from the posterior margin of tergite IX to the tip of gonocoxite. The measurements were given only for undamaged structures. Drawings were completed by tracing the photographs. Drawings (Figs. 1A,B,D and 3B) and photographs were made by Iwona Kania-Kłosok. Map was built using the map Maps-For-Free (https://maps-for-free.com) and modified with the software programs Corel Draw and Corel Photopaint X7. The wing venation and male genitalia nomenclature follows that of^17,26,27^.

## Systematic palaeontology

Order Diptera Linnaeus, 1758^28^.

Infraorder Tipulomorpha Rohdendorf, 1961^29^.

Family Limoniidae Speiser, 1909^30^.

Genus *Helius* Lepeletiere & Serville, 1828^31^.

Type species: *Helius longirostris* (Meigen, 1818)^32^.

### Key to extinct species of the genus *Helius* known from Cretaceous

1. Rostrum very elongate reaching almost 0.3 the body length ……………………………………………………………… *Helius* (*Helius*) *ewa* Krzemiński, Kania & Azar, 2014^5^
  − Rostrum distincly shorter than 0.3 the body length … 2.
2. Rostrum elongate, but as long as or shorter than head …*Helius krzeminskii* Ribeiro, 2002^7^.
  ……………………………………………………………… Rostrum longer than head … 3.
3. Palpus shorter than rostrum … *Helius botswanensis* Rayner & Waters, 1990^6^.
  ……………………………………………………………… Palpus as long as or longer than rostrum … 4.
4. Palpus as long as rostrum, last palpomere not very elongate approximately as long as second and third, penultimate palpomere very small and short, about 0.25 the length of the fourth palpomere; antennomeres 1–4 relatively massive … *Helius hispanicus* sp. nov.
  − Palpus longer than rostrum, last palpomere elongate longer second and third; penultimate palpomere elongate, palpomeres 3 elongate, at least 0.3 the length of the fourth palpomere; antennomeres 1–4 elongate, cylindrical ……………………………………………………………… 5.
5. Last palpomere as long as the preceding all taken together …6.
  − Last palpomere longer than the preceding all taken together ………………………………………………………………7.
6. Flagellomeres elongate and tiny, slender, last flagellomere distinctly narrow, as long as penultimate one; palpus almost equal in length to rostrum … *Helius* (*Helius*) *alavensis* Kania, Krzemiński & Arillo, 2016^2^.
  − Flagellomeres cylindrical, last flagellomere longer than penultimate one; palpus almost two times longer than the length of rostrum *Helius lebanensis* Kania, Krzemiński & Azar, 2013^4^.
7. Gonocoxite with extra lobe at the apex on dorsal surface, relatively wide and elongated, of comparable size to outer and inner gonostylus, only slightly shorter than outer and inner gonostylus … *Helius* (*Helius*) *spiralensis* Kania, Krzemiński & Arillo, 2016^3^.
  − Lacks extra lobe of gonocoxite … *Helius turolensis* sp. nov.

### *Helius turolensis* sp. nov.

(Figs. 1, 2).

**Figure 2:**
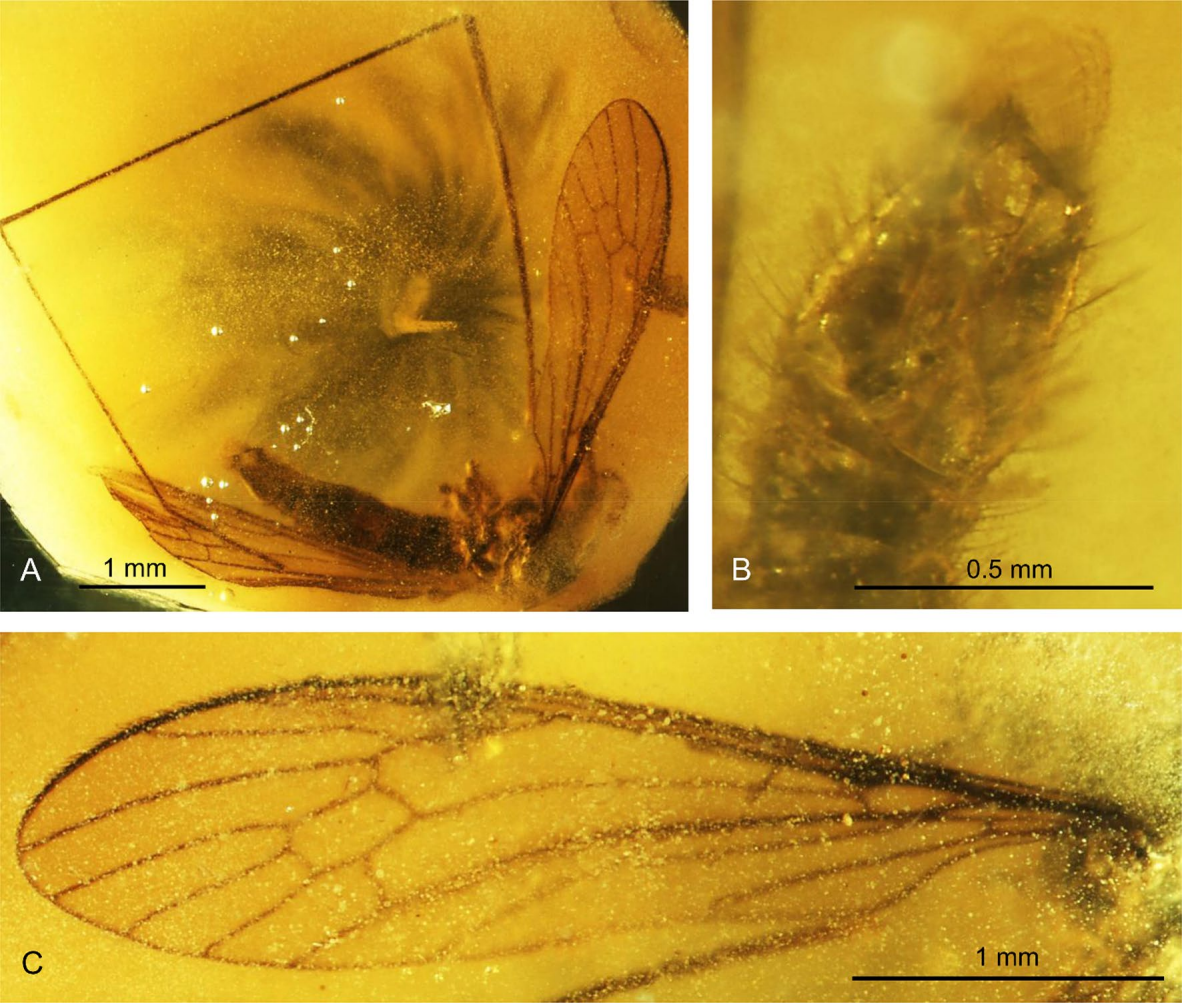
*Helius turolensis* sp. nov. No. 53–10-54 (male), holotype. (**A**) body, latero-ventral view; (**B**) hypopygium; (**C**) wing.

#### Diagnosis

Rostrum only slightly longer than head, constitute 0.5 the length of head; antenna relatively slender and short, terminate just beyond the head, each flagellomere with two very elongate setae, approximately 6.7 times longer than length of segments bearing them; palpus longer than rostrum; last palpomere elongate, slender, longer than palpomeres 1–3 combined; third palpomere widened in basal part, about 0.3 × the length of the fourth palpomere.

#### Etymology

The specific epithet is derived from Teruel, where the specimen was been found.

#### Material examined

Holotype No. SJ-10-54 (male), housed at the Fundación Conjunto Paleontológico de Teruel-Dinópolis (Teruel, Spain).

#### Horizon and locality

The specimen was found in amber from gray-black claystones with abundant plant remains which corresponds to a deposit of a fluvial deltaic swamp, in the Utrillas Group^33^, Lower Cretaceous, upper Albian.

The outcrop of San Just^34^ is located in the Maestrazgo Basin, municipality of Utrillas (Province of Teruel, Aragón Autonomous Community, eastern Spain).

#### Description

Body (Fig. 2A) pale brown, 2.97 mm long.

*Head* (Figs. 1A, 2A): 0.36 mm wide, 0.30 mm high; rostrum 0.38 mm long; antenna (Figs. 1A, 2A) 16-segmented, about 0.67 mm long (Fig. 1C); scape cylindrical and not very elongate, widened in apical part, pedicel short and rather wide, approximately as long as wide; first flagellomere cylindrical, fraction longer than the next one, not extended at the base, three basal segments of antenna with not very elongate a few setae, longer than width and length of segments bearing them, flagellomeres became more slender to the apex of antenna; flagellomeres 2–14 with very long setae, elongate setae 0.51 mm long. Maxillary palp (Figs. 1A, 2A) four segmented, 0.51 mm long (1/0.05 mm; 2/0.07 mm; 3/0.12 mm; 4/0.27 mm) slender, two basal segments almost equal in length, only slightly widened at distal part, third palpomere massive, longer than first and second palpomeres, but shorter than first and second palpomeres combined, widened at basal part and narrowed at distal part, fourth palpomere very elongate, longer than all other palpomeres arranged together, tiny.

*Thorax* (Fig. 2A): wing (Figs. 1B, 2A,C) 3.65 mm long, 0.93 mm wide; pterostigma present, oval, pale-brown; Sc relatively short, ends well before fork of Rs; Rs relatively short, R_2+3+4_ almost 1.5 × the length of Rs; R_1_ ends opposite 0.60 × length of R_2+3+4_ level; R_5_ longer than R_2+3+4_; cross-vein m-cu connected with M_3+4_ behind half of its length measured from fork of Mb; d-cell, twice longer than wide; M_3_ slightly waved, 1.5 × as long as d-cell; A_1_ and A_2_ slightly waved, arched at the margin of wing, A_1_ elongate. Tip of A_1_ behind Rb bifurcation level.

Abdomen: hypopygium (Figs. 1D, 2A,B): 0.66 mm long, with gonocoxite relatively narrow and elongate, approximately 3 × as long as wide; at the apex of gonocoxite bunch of very elongate setae arranged around the tip; outer and inner gonostyles of comparable size; outer gonostylus elongate, slightly widened in the distal part.

#### Comparison

*Helius turolensis* sp. nov. differs from all other Cretaceous representatives of genus by the ratio between the length of rostrum, antenna, palpus and head.

This species differs from species known from the Eocene and the Miocene periods by tiny, slender antennae with characteristic, very elongate setae on each flagellomere, 6.7 × longer than length of segments bearing them.

Moreover, in contrast to *H. hispanicus* sp. nov. third palpomere is massive and widened at the base, in *H. turolensis* sp. nov. this palpomere is very small, not longer than 1.5 × its width.

The antennae of *H. turolensis* sp. nov. bears two very elongate, symmetrically arranged setae, similarly to *H. hispanicus* sp. nov. In contrast to *H. hispanicus* sp. nov. in *H. turolensis* sp. nov. palpus is more slender, last palpomere is elongate and tiny, longer than the length of all other palpomeres combined in *H. hispanicus* sp. nov. last palpomere is not very elongate approximately as long as second and third combined. Differences are also well visible in ratio between the length of rostrum, antenna, palpus and head between these two species.

In *H. turolensis* sp. nov. palpus is longer than rostrum, antenna is almost 0.25 × longer than palpus, while in *H. hispanicus* sp. nov. palpus and rostrum are in length, antenna is almost 0.14 × longer than palpus.

In *H. alavensis*, antennae are slender, without very elongate setae as in *H. turolensis*. Palpus in *H. alavensis* is only slightly longer than rostrum, approximately 1.14 × the length of rostrum, in *H. turolensis* 1.25 × . In contrast to *H. spiralensis*, gonocoxite in *H. turolensis* lacks extra lobe, in *H. spiralensis* the extra lobe is present and bears elongate, strong setae at apex.

In contrast to other Cretaceous species *H. turolensis* differs especially by morphology of the head, the wing venation and the male genitalia.

In *H. ewa*, rostrum is very elongate, reaching about the third of body length, in *H. turolensis* rostrum is only slightly longer than head, head ca. 0.36 mm long; rostrum ca. 0.38 mm long. In *H. lebanensis*, discal cell is opened, in contrast to *H. turolensis* where discall cell is closed. In *H. krzeminskii*, rostrum is relatively short and the differences between these two species are well visible comparing the ratio of the length of rostrum, the length of head and head appendages. In *H. botswanensis* palpus, is shorter than rostrum, rostrum is approximately twice as long as head, while in *H. turolensis*, palpus is longer than rostrum, rostrum is approximately as long as the head.

### *Helius hispanicus* sp. nov.

(Figs. 3, 4).

**Figure 3:**
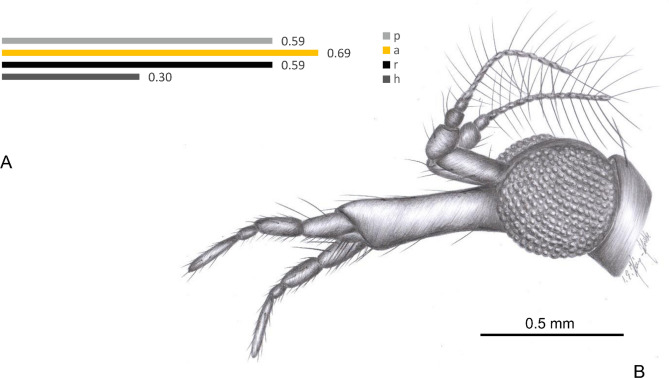
*Helius hispanicus* sp. nov. No. MCNA 9946 (sex undefinied), holotype. (**A**) the diagram illustrating the relationship between the length of rostrum (r), antenna (a); palpus (p) and head (h); (**B**) head. Abbreviation of head (h): as in Fig. 2.

**Figure 4:**
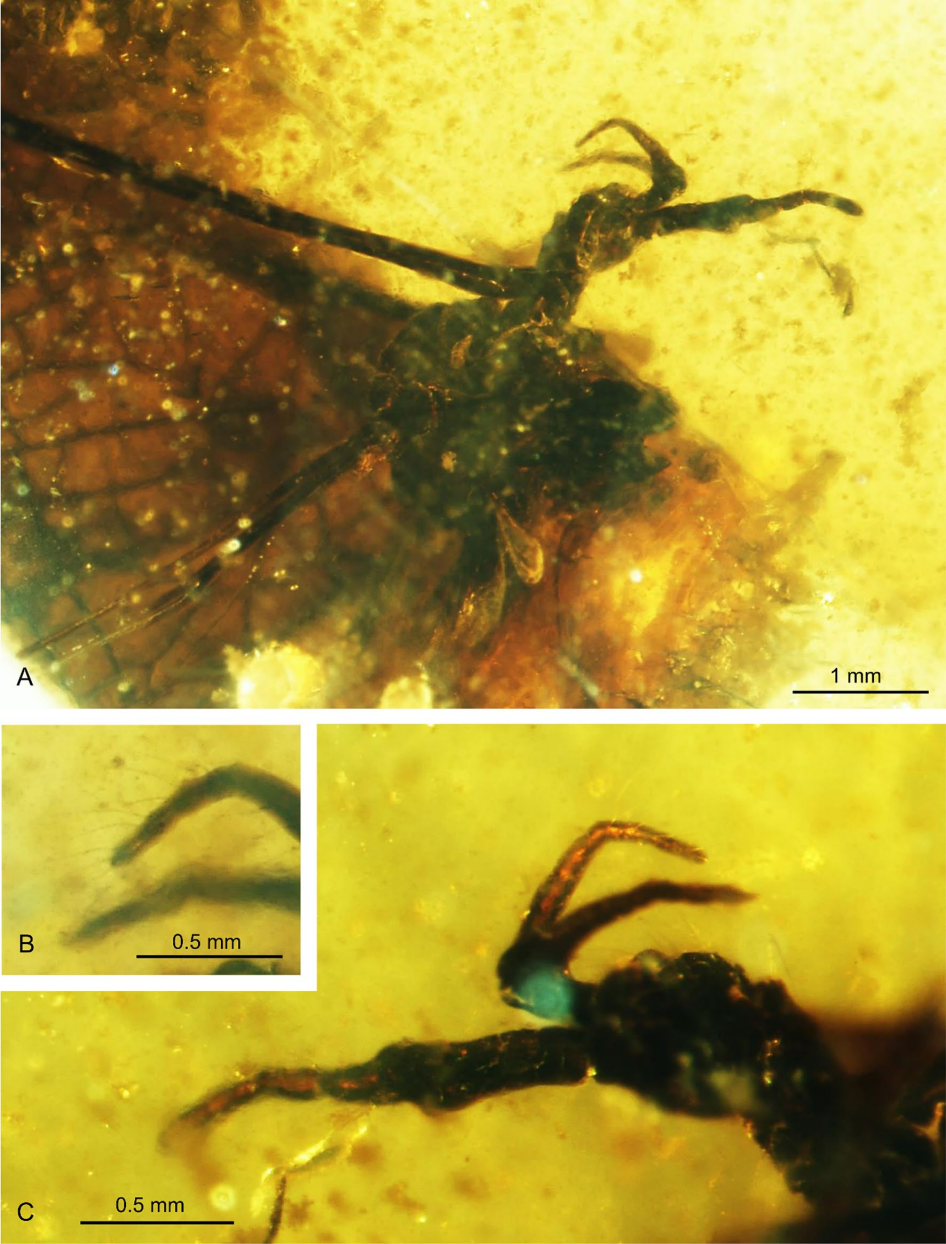
*Helius hispanicus* sp. nov. No. MCNA 9946 (sex undefinied), holotype. (**A**) fore part of the body (lateral view); (**B**) last palpomeres; (**C**) head (lateral view); Abbreviation of head (h): as in Fig. 2.

#### Diagnosis

Rostrum only slightly longer than head; antennomeres 1–4 relatively massive and short, terminate just beyond the head; each flagellomere with two very elongate setae, approximately 4.5 × longer than segments bearing them; palpus as long as rostrum; last palpomere not very elongate approximately as long as second and third; penultimate palpomere very small and short, about 0.25 × the length of the fourth palpomere.

#### Etymology

The specific epithet is derived from *Hispania*, Latin name for Spain.

#### Material examined

Holotype No. MCNA 9946 (sex unidentified), Peñacerrada, Álava, Spain, housed at the Museo de Ciencias Naturales de Álava, Vitoria, Spain.

#### Horizon and locality

The type specimen was found in amber from coal levels with abundant plant remains developed in delta plain areas which corresponds to the top of filling sequences of interdistributary bays but it is also found in filling deposits of abandoned fluvial channels or crevasse splay, in the Utrillas Group^33^, Lower Cretaceous, upper Albian.

The outcrop of Peñacerrada I^34^ is located in the Basque-Cantabrian Basin, municipality of Moraza (Province of Burgos, Castilla y León Autonomous Community, northern Spain).

#### Description

The head and thorax (Figs. 3B, 4A) brown.

*Head* (Figs. 3, 4A,C): 0.30 mm wide, 0.33 mm high; rostrum 0.59 mm long; antenna (Figs. 3B, 4A,C) 16-segmented, about 0.69 mm long (Fig. 3A) (1/0.43 mm; 2/0.22 mm; 16/0.10 mm); scape cylindrical, but wide and massive, elongate, 2.5 × longer than wide, with a few not very elongate setae; pedicel relatively elongate and wide, massive, widened in distal part, longer than wide; first flagellomere wide, relatively short, flagellomeres 1–14 tapering toward apex; each flagellomere with two symmetrically arranged very long setae, much longer than segments bearing them, last flagellomere with two very elongate setae arranged at the tip since the last it’s the one at the tip. Maxillary palp (Figs. 3B, 4A–C) four-segmented, two basal segments massive, relatively elongate and almost equal in length (length of palpomeres 0.59 mm: 1/0.14; 2/0.14 mm; 3/0.07 mm; 4/0.24 mm), third palpomere small, 1.5 × longer than wide, about half the length of first and second palpomeres; last palpomere not very elongate, shorter than all other palpomeres combined, only 4 × as long as wide.

#### Remarks

The specimen is poorly preserved. Abdomen not preserved, wings and legs are only partly preserved. But, the relation between the length of rostrum, antennae and palpus, and other morphological features like morphology of antenna or palpus allow to classify this specimen to the genus *Helius* and to describe it as a new species.

#### Comparison

*Helius hispanicus* sp. nov. differs from all other representatives of genus by the by the occurrence of very small and short third palpomere, which constitute only 0.25 × the last one. Third palpomere is very small, not longer than 1.5 × of its width, while in all other extinct species known from Cretaceous period this segment of maxillary palpus is rather elongate and widened. Moreover, *Helius hispanicus* sp. nov. differs from Cretaceous representatives of genus by ratio between the length of rostrum, antenna, palpus and head. In *H. spiralensis* and *H. alavensis*, palpus is longer than rostrum, in *H. hispanicus* is as long as rostrum, in *H. botswanensis* is distinctly shorter than rostrum. Rostrum as long as or shorter than head occur in *H. krzeminskii*, in *H.* (*H.*) *ewa* rostrum is very elongate reaching almost 0.3 the body length, while in *H. hispanicus* rostrum is longer than head, but and shorter than in *H.* (*H.*) *ewa.*

On the antennae of this species occur two very elongate, symmetrically arranged setae, these very elongate setae does not occur in other species of *Helius* known from fossil record of younger periods like Eocene.

## Discussion

**Figure 5:**
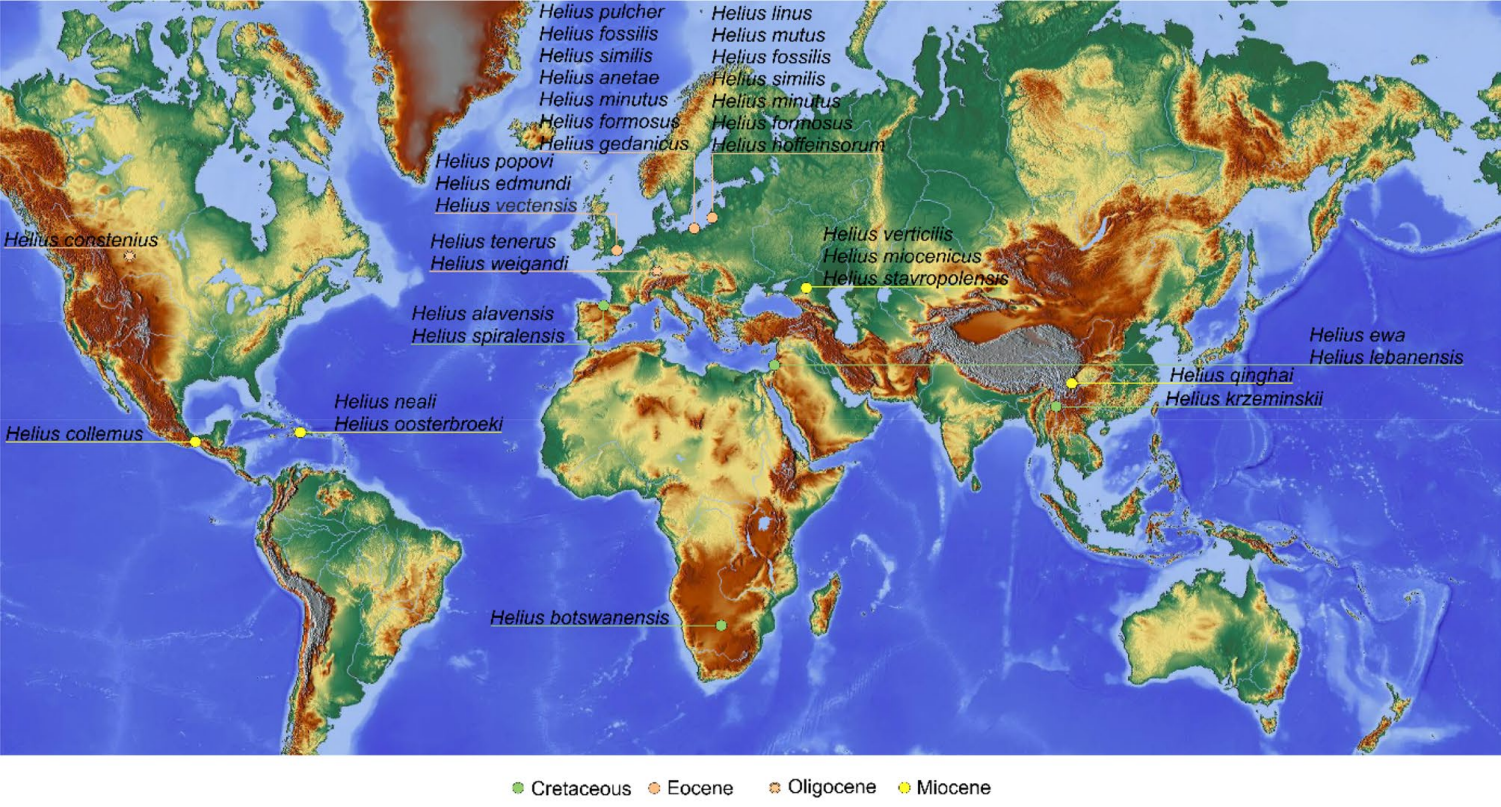
Maps with enlarged view of the distribution of know Cretaceous localities of the representatives of the genus *Helius*. Map was built using the map Maps-For-Free (https://maps-for-free.com) and modified with the software programs Corel Draw and Corel Photopaint X7.

Six species of flies belonging to the genus *Helius* are known from the cretaceous period and only three are known from Spanish amber^1–3^ (Fig. 5). All the species of the genus *Helius* described so far on the basis of inclusions in Cretaceous Spanish amber came from the same outcrop Peñacerrada I, Álava, Basque-Cantabrian Basin, Spain.

One of the species new to science included in this work, *H. hispanicus* sp. nov., was described on the basis of inclusions also originating from the outcrop Peñacerrada I, Álava, while the other fossil representative *H. turolensis* sp. nov. was found at the outcrop of Sun Just located in the Maestrazgo Basin. Both the first and second positions are dated to upper Albian. It should be noted that this is the first record of the genus *Helius* from fossil record of the Maestrazgo Basin.

Most of the 150 Iberian Penisula amber deposits are dated to Albian (Early Cretaceous), only a few of these localities are dated to Late Cretaceous e.g. in Asturias or Catalonia, and only two localities with amber are dated to the late Triassic (both in Alicante)^35^.

Both Basque-Cantabrian Basin and Maestrazgo Basin were formed during significant changes in fauna and flora on earth. The large sedimentary Basque-Cantabrian Basin has developed between the Iberian and European tectonic plates, the Maestrazgo basin is an intercontinental basin located in the Iberian Range^35^. In the Basque-Cantabrian Basin amber is preserved in rocks rich in organic matter from sediments, is large quantity of plants remains, coal and other continental organic material which was transported by rivers. Largest deposits of Maestrazgo Basin is San Just with abundant plant remains and fusinished wood, were probably formed during subtropical hot-humid environment with hot and dry areas^35^.

It is in such an area with a subtropical, hot and humid environment and dry areas that the representatives of the genus *Helius* occurred in Cretaceous period.

Recent species of the genus *Helius* occur mainly in Oriental, Australian and Oceanian or Neotropic regions^21^ and many species of this genus prefer rather hot climates.

Based on geological and biotic evidence, a sharp distinction between the northern territories of Laurasia and the southern landmasses of Gondwana had happened in the earliest late Mesozoic^36^. According to Eurogondwana model, which explains the migration of Eurogondwanan lineages to Laurasia^36^, European region were probably connected to Africa during the Early Cretaceous through the Apulian microplate and was served 130 Ma^37,38^. The research conduct that e.g. the hormurid scorpions colonized Laurasia from Africa via the Apulia microplate (Europa terrane) in the Cretaceous. The migrations of some taxa into Asia and North America were possible through Europe, on the other hand, Europe was preventing Laurasia taxa from migrating to Africa^38^.

Most species of the genus *Helius*, whose bodies have been preserved in Spanish amber, have certain common features. Antennae and palpi are usually thin, very narrow, slender, the flagellomeres are small and the boundaries between them are often difficult to distinguish. The antennae of species known from Spanish amber are also relatively short and among the species of the genus *Helius* we do not find representatives with a very elongate rostrum, the rostrum is usually only slightly longer than the head, as in *Helius alavensis* or *Helius spiralensis*. These set of characters distinguish the group of species known from Spanish amber from other known fossil species. A comparable long rostrum also occurs in a bizarre stem scydmaenine beetle from the mid-Cretaceous Burmese amber^39^, which represents a case of convergent evolution in Cretaceous insects.

These insects rapidly evolved during the Cretaceous period and adapted to the new food spectrum offered by angiosperms (flowering plants) that becoming abundant at that time^2–5,7,40,41^, but as it is suggested^42^, some of them could drops of extinct gymnosperms to feed on floral nectar and pollination.
